# Recent Advances in Fire-Retardant Silicone Rubber Composites

**DOI:** 10.3390/polym16172442

**Published:** 2024-08-28

**Authors:** Yi-Hao Tang, Jun Liu, Zuan-Yu Chen, Yang Li, Cheng-Fei Cao, Guo-Dong Zhang, Long-Cheng Tang

**Affiliations:** 1 China Helicopter Research and Development Institute, Jingdezhen 333001, China; 2 College of Materials Science and Chemical Engineering, Harbin Engineering University, Harbin 150001, China; 3 Key Laboratory of Organosilicon Chemistry and Material Technology of MoE, Key Laboratory of Silicone Materials Technology of Zhejiang Province, College of Material, Chemistry and Chemical Engineering, Hangzhou Normal University, Hangzhou 311121, China; 2022112009029@stu.hznu.edu.cn (J.L.); chenzuanyu@stu.hznu.edu.cn (Z.-Y.C.); ly@hznu.edu.cn (Y.L.); chengfeicao168@gmail.com (C.-F.C.); zhangguodong@hznu.edu.cn (G.-D.Z.)

**Keywords:** silicone rubber, composite, intrinsic flame retardation, additive-type flame retardation, flame retardancy, mechanical and thermal properties

## Abstract

Silicone rubber (SR), as one kind of highly valuable rubber material, has been widely used in many fields, e.g., construction, transportation, the electronics industry, automobiles, aviation, and biology, owing to its attractive properties, including high- and low-temperature resistance, weathering resistance, chemical stability, and electrical isolation, as well as transparency. Unfortunately, the inherent flammability of SR largely restricts its practical application in many fields that have high standard requirements for flame retardancy. Throughout the last decade, a series of flame-retardant strategies have been adopted which enhance the flame retardancy of SR and even enhance its other key properties, such as mechanical properties and thermal stability. This comprehensive review systematically reviewed the recent research advances in flame-retarded SR materials and summarized and introduced the up-to-date design of different types of flame retardants and their effects on flame-retardant properties and other performances of SR. In addition, the related flame-retardant mechanisms of the as-prepared flame-retardant SR materials are analyzed and presented. Moreover, key challenges associated with these various types of FRs are discussed, and future development directions are also proposed.

## 1. Introduction

**Figure 1:**
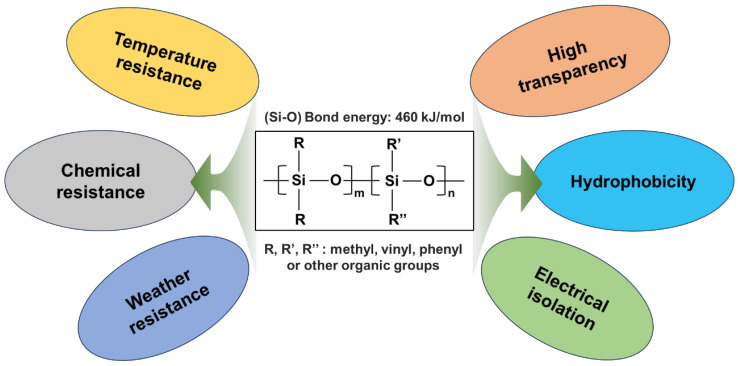
Molecular structure of silicone rubber and its unique properties.

Silicone rubber (SR), a widely recognized advanced elastomer, has gained significant academic and industrial attention in recent decades [1,2,3,4]. The special organic–inorganic hybrid molecular structure endows the SR with exceptional properties, e.g., high- and low-temperature resistance, weathering resistance, chemical stability, and electrical isolation [5,6]. Furthermore, it also possesses hydrophobicity and good transparency (Figure 1). Owing to these attractive physical properties, SR materials have been applied in many key areas of construction, transportation, electronics, the auto industry, aviation, and biology [7,8,9,10,11,12]. However, pendant organic groups, e.g., methyl and vinyl, of the SR molecular chain render it intrinsically flammable once it encounters a flame attack [13,14,15], which severely restricts its practical application in fields with stringent flame-retardant standards. Therefore, how to effectively improve its fire retardancy is a key issue for broadening its practical applications.

So far, there are two common strategies to fabricate flame-retardant SR, i.e., changing the structure of SR chains and adding flame retardants [16]. The former involves the modification of the molecular chain, which is achieved by introducing flame-retardant units containing special elements, e.g., phosphorus and nitrogen [17,18]. The latter involves the incorporation of flame retardants or flame-retardant fillers into the SR matrix. There are many types of additive-type flame retardants, e.g., halogen-containing flame retardants [19,20], phosphorus-/nitrogen-/silicon-containing flame retardants [21,22,23,24], inorganic flame retardants [25,26,27], intumescent flame retardants [28,29,30], nanofillers or nano-flame retardants [31,32,33], etc. Therefore, each of them has its advantages and shortcomings. For example, halogen-containing flame retardants can largely enhance the flame retardancy of SR; however, they have a significant ecological bioaccumulation issue and release toxic smoke when burned, which has been receiving considerable attention [19]. Nano-flame retardants can enhance flame retardancy and maintain the mechanical properties of SR, but their poor dispersion in the polymer matrix is a problem to be tackled [34,35,36]. Overall, it is vital to develop highly efficient flame-retardant systems and fabricate high-performance flame-retardant SR.

Indeed, a series of flame-retardant SR materials have been reported in recent years [37,38,39,40,41]. However, based on the investigation of recent review of the flame-retardant SR [16], it is demonstrated that there is lack of critical review on the advancements of the flame-retardant SR material systems in the past decade. Herein, this comprehensive review will introduce the two main strategies for SR, i.e., intrinsic flame retardation and additive-type flame retardation, and will mainly focus on the synthesis and modification of various flame-retardant additives and their influences on the flame-retardant performance and other properties of SR, followed by the corresponding flame-retardant mechanisms. Therefore, the key challenges and future research directions associated with those flame-retardant systems are also presented.

## 2. Thermal Decomposition Behavior of SR

**Figure 2:**
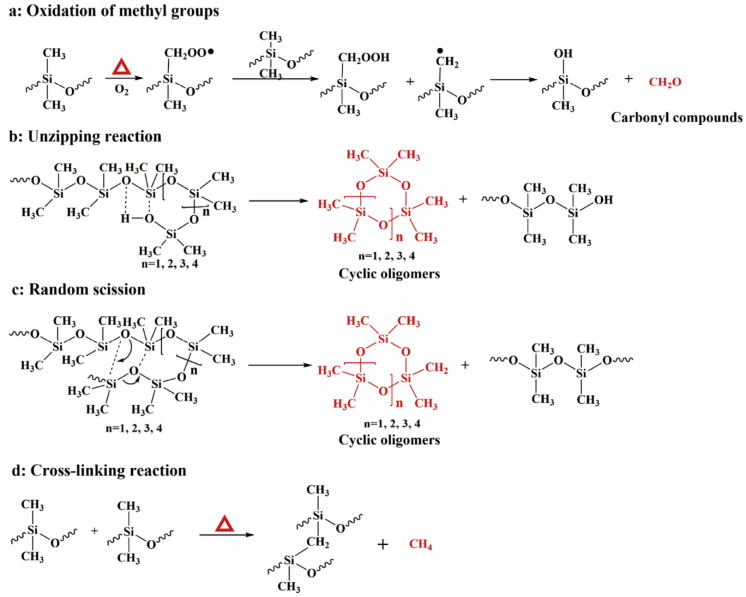
Thermal degradation mechanism of SR under air atmosphere. Reproduced with permission from [24]. Copyright 2017, Elsevier.

Camino et al. first systematically investigated and reported the thermal decomposition behaviors of SR [42,43], as shown in Figure 2. Specifically, thermal degradation involves a series of complicated reactions, i.e., the decomposition of the lateral organic groups, the depolymerization of the Si-O backbone, and cross-linking reaction [15,24,44].

The dissociation energy of Si-O is ~108 kcal/mol, higher than that of Si-C bond (~78 kcal/mol). Thus, the first step of thermal degradation of SR involves the oxidation of organic groups. More specifically, the methyl groups of the SR side chains are oxidized to peroxide and then react with Si-CH_3_, further leading to the formation of silanol groups and (Si-OH) and the release of methanal [45]. When further increasing the temperature, the unzipping reaction of Si-OH groups and the random scission reaction of the backbone occur, and the cyclic oligomers are generated. Meanwhile, methane is generated due to the cleavage of Si-CH_3_ [42], and the cross-linking reaction of the macro-radicals decreases the flexibility of the SR chain [43].

## 3. Laboratory Fire Testing Methods

To test the flame-retardant properties of materials and evaluate their fire risk levels, a series of fire testing methods have been established. Therefore, corresponding test standards have also been set. At present, the most common and widely used test methods for SR include the UL 94 vertical burning test, the limiting oxygen index (LOI) test, and the cone calorimeter test [35,46].

### 3.1. UL 94 Vertical Burning Test

UL 94 tests are quick and simple, requiring only small samples. For SR materials, the vertical burning test is usually used to evaluate their combustion behavior and flame retardancy. The UL94 rating of the sample can be clarified based on the measured parameters. Furthermore, the classification of materials also considers whether flaming melt dripping occurs. According to the standard IEC 60695-11-10 [47], it requires five parallel samples in each measurement. Table 1 contains the criteria for various UL 94 ratings.

### 3.2. Limiting Oxygen Index Test

The limiting oxygen index (LOI) is defined as the minimum oxygen concentration in the nitrogen–oxygen mixture that can maintain flame burning of the sample for 180 s or consume the sample for a 5 cm length. This parameter is assessed according to ASTM D2863 and ISO 4589 standards [48]. During the test, the sample is vertically placed in a controlled atmosphere and ignited, and its burning behavior is observed. The LOI value is determined by gradually reducing the oxygen concentration until combustion ceases. For greater accuracy, the average LOI value is calculated from five specimens per batch. Generally, a higher LOI value indicates better flame retardancy. Since the ambient air contains approximately 21 vol% oxygen, materials with an LOI below 21% are considered “flammable”, while those with higher values are deemed “self-extinguishing”. The calculation formula for LOI is:

(1) $$LOI\left(\%\right)=\frac{\left[O_{2}\right]}{\left(\left[O_{2}\right]+\left[N_{2}\right]\right)}\times 100\%$$

[O_2_] and [N_2_] stand for the flow rates of O_2_ and N_2_, respectively.

### 3.3. Cone Calorimeter Test

Among those fire-retardant test methods, the cone calorimetry test is the most reliable and effective method that can simulate the combustion behaviors of materials in real fire scenarios by using medium-sized samples in the laboratory [49,50]. According to international standards (e.g., ISO 5660), a cone heater is used to deliver radiant heat at a fixed heat flux to a specimen [51]. Upon ignition, the sample starts to burn and releases heat, smoke, and other combustion products. During the whole combustion process, related heat data are collected, including the heat release rate (HRR), total heat released (THR), etc. Meanwhile, the smoke data, e.g., the total smoke production (TSP) and total smoke released (TSR), can also be determined. As a result, the flammability of the samples can be quantitatively evaluated based on those heat and smoke parameters.

## 4. Flame Retardance of SR

At present, two typical strategies are adopted to enhance the flame retardancy of SR, i.e., intrinsic flame retardation and additive-type flame retardation. The former involves a chemical reaction or modification by incorporating some flame-retardant units or groups containing specific elements (e.g., N, P, S, B and Si) into polymer chains of SR, thereby preparing the intrinsic flame-retardant SR. The additive-type flame retardation is achieved by adding flame retardants or flame-retardant fillers into the SR matrix. Generally, synergistic flame retardants, e.g., boron nitride (BN), carbon nanotubes (CNTs), graphene and its derivatives, montmorillonite (MMT), etc., can also be adopted to further enhance the flame-retardant efficiency of the above two types of flame retardation, as the introduction of synergistic flame retardants can generally produce multiple flame-retardant mechanisms, i.e., gas-phase and condense-phase flame-retardant mechanisms (which will be discussed later). Therefore, these two approaches have their advantages and disadvantages. Intrinsic flame-retarding polymers generally possess good comprehensive performance, e.g., flame retardancy and mechanical properties, but they usually involve a series of complex modification steps, and the related synthesis processes are usually time-consuming and have high costs. By comparison, the fabrication technology of additive-type flame retardation is simple, and the flame-retardant performance of materials can be adjusted easily. Therefore, a major drawback is that the mechanical properties of material matrices are often largely deteriorated due to the high addition of flame retardants. In the following section, a series of flame-retardant SR material systems based on various flame-retarding approaches will be introduced and discussed. It should be noted that, based on previously reported studies and their practical applications in SR materials, intrinsic flame retardation is seldom investigated, whereas additive-type flame retardation has been widely studied and applied. Thus, the following sections mainly focus on the advancements in additive-type flame retardation of SR. Furthermore, considering the limited applications of the halogen-containing flame retardant system, such systems will not be introduced.

### 4.1. Intrinsic Flame Retardation

Intrinsic flame retardation has garnered considerable attention owing to the good comprehensive performance of intrinsically flame-retardant polymers. Such polymers are typically synthesized by copolymerizing existing polymers with flame-retardant units or incorporating these units as side groups along the polymer chain [52,53]. Compared to equivalent additive flame retardants, flame-retardant units grafted onto the molecular chain exhibit a more uniform distribution within the polymer matrix, resulting in higher flame-retardant efficacy than traditional additive flame retardants. For SR, based on the structure characteristics of prepolymers and the cross-linking reaction of SR, the flame-retardant units can be grafted onto prepolymer side chains and further become part of the SR polymer matrix via the cross-linking reaction.

**Figure 3:**
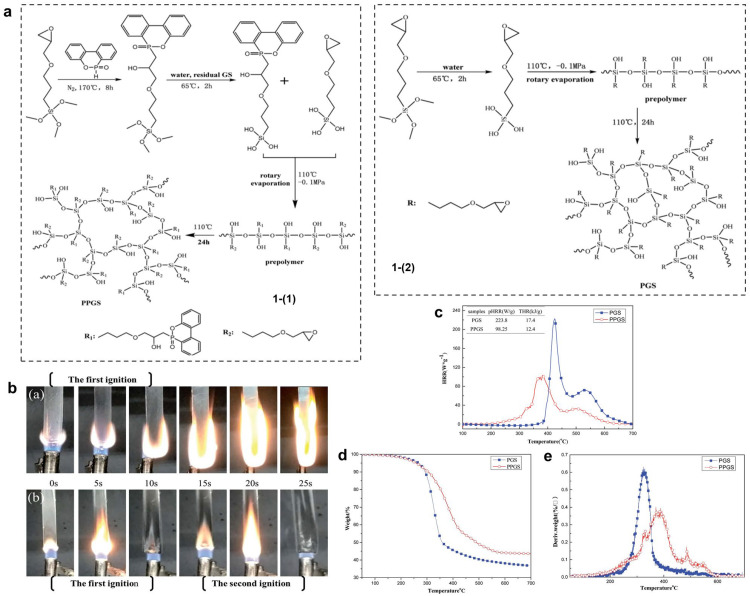
(**a**) Synthesis route of PPGS (1-1) and PGS (1-2). (**b**) The combustion process of (a) PGS and (b) PPGS. (**c**) HRR curves of PGS and PPGS. (**d**) TG and (**e**) DTG curves of PGS and PPGS in air atmosphere. Reproduced with permission from [18]. Copyright 2017, The Royal Society of Chemistry.

To overcome the limitations of the SR systems with additive-type flame retardants, e.g., the deterioration in the mechanical performance, transparency, and elasticity, Chen and co-authors designed and prepared an intrinsic flame-retardant SR containing a phosphaphenanthrene structure [18]. In this work, the typical reactive phosphorus compound (DOPO) was employed to react with the siloxane monomer to synthesize the intrinsic flame-retardant SR (PPGS) (see Figure 3). For comparison, the PGS and unmodified SR were also prepared (see Figure 3a). The related results showed that the PPGS showed better flame retardancy, e.g., achieving a UL94-V0 rating and a high LOI of ~42%, as well as a large decrease in HRR and THR when compared with PGS. Therefore, the condensed-phase flame-retardant action based on the coupling between the phosphaphenanthrene group and siloxane was revealed by the authors. Moreover, it is worth noting that both PPGS and PGS showed high transparency compared to the light-proof SR added with DOPO powder.

For certain application scenarios, e.g., thermal protection of structural units of spacecraft, it is required that the service materials possess flexibility, anti-ablation, and sealing performance [3,54]. Thus, there is a huge need for flexible ablation materials (FAMs) with good elasticity and deformability, as such materials are highly effective in protecting complex, deformable, and dynamic structures. SR is an ideal material matrix for FAMs due to its excellent flexibility, thermal oxidation resistance, and thermal stability. However, under high-temperature heat flux, the char layer of pure SR is brittle; thus, to form a dense and robust char layer during ablation, fiber fillers [55,56] and powder fillers [57] are added to the pure SR matrix.

Currently, direct physical blending is the primary method for introducing hetero-elements (such as B, Zr) into FAMs [58,59], but the direct incorporation method cannot achieve a uniform dispersion of hetero-elements due to the aggregation of the fillers during preparation. A previous study indicated that a hetero-element, i.e., Zr, can be introduced into the silicone resin matrix by molecular modification, thus achieving a homogeneous distribution of Zr and high thermal oxidation resistance of the as-prepared zirconium-silicone resin [58]. With this in mind, Tian and co-authors designed and obtained an interpenetrating double network (Zr-PDMS), i.e., a polysiloxane network and a Zr-containing network, within the SR matrix based on hydrosilylation and hydrolysis–condensation processes [17]. The mechanical test results demonstrated significantly increased mechanical strength, offering exceptional advantages for the SR matrix in terms of adapting to large deformations. Most notably, the introduction of Zr significantly enhanced the char layer’s integrity and coherence with the virgin matrix, resulting in superior anti-ablation performance. Consequently, the Zr-PDMS showed exceptional ablation resistance. Based on the enhanced flexibility and ablation resistance, such materials can be applied as highly ablation-resistant FAMs in the aerospace field. Zhang et al. developed a boron-hybridized SR material (named VP-PBSi/SR) exhibiting excellent thermal stability and good mechanical properties [60]. The vinylphenyl-functionalized polyborosiloxane (VP-PBSi) was synthesized via the sol–gel method by reacting boric acid (BA), phenyltriethoxysilane (PTEOS), and triethoxyvinylsilane (VTEOS). The results demonstrated a significant enhancement in the thermal stability and mechanical properties of the SR, e.g., the tensile strength and char yield reached 4.65 MPa, and 72.3%, respectively, representing increases of 20.2% and 26.1% compared to neat SR.

### 4.2. Additive-Type Flame Retardation

For additive-type flame retardation, flame retardants or flame-retardant fillers are typically added into SR via physical mixing [61]. The primary advantage of this method is its simplicity in achieving flame retardancy in polymers. For liquid SR prepolymers, additive flame retardants are usually incorporated through a straightforward mixing process. However, for SR prepolymers with high molecular weight or high viscosity, the incorporation of flame retardants often involves the use of a two-roll mixing mill. It should be noted that the selection of a flame-retardant system needs to be matched to the processing temperature of the SR. For example, for an SR with a vulcanization temperature of beyond 150 °C, to prevent the decomposition of flame retardants during processing, it is required to select flame retardants with decomposition temperatures beyond the processing temperature of SR. So far, based on the reported studies and practical application cases of SR, phosphorus/nitrogen/silicon flame retardants, inorganic flame retardants, intumescent flame retardants, and nano-flame retardants have been employed to prepare flame-retardant SR composites. In the following section, SR systems with various flame retardants or flame-retardant fillers will be presented and systematically introduced.

#### 4.2.1. Phosphorus-/Nitrogen-/Silicon-Containing Flame Retardants

Phosphorus-based flame retardants are considered effective halogen-free flame retardants [62]. They can produce condensed- and gas-phase flame-retardant actions simultaneously. In the condensed phase, the released polyphosphoric acid can promote the dehydrated carbonization of the polymer matrix, creating a physical barrier effect. Meanwhile, in the gas phase, free radicals generated from phosphorus-based flame retardants, such as PO·, PO_2_·, and HPO, react with free radicals produced by the polymers. This interaction further hinders the free-radicals-maintained combustion [53,63]. In terms of SR, it is difficult to achieve good flame retardancy by adding single phosphorus-based flame retardants. Usually, phosphorus-containing flame retardants have poor thermal stability and water resistance, while the application scenarios of SR may involve high-temperature and high-humidity environments. In addition, they also exhibit poor heat resistance during combustion; therefore, the char residue of SR is often porous and loose, rendering it ineffective in condensed-phase flame retardation [22]. Unlike phosphorus-based flame retardants, the flame-retardant action of the nitrogen-containing flame retardants mainly involves the gas phase. When the temperature reaches the decomposing temperature, they begin to decompose into small derivatives and produce non-combustible gases, e.g., NH_3_. This process absorbs a large amount of heat, thereby decreasing the surface temperature of the polymer substrate [50,64]. Additionally, the generated nonflammable gases can dilute the concentration of flammable vapor products released from the polymer and the surrounding oxygen [65].

Zhou and co-authors utilized melamine cyanurate (MCA) to develop flame-retardant SR and investigated the flame retardancy and thermal aging properties of the SR composites [66]. The results indicated that the introduction of MCA slightly reduced the flame retardancy of SR. However, the residual mass increased by ~10.0% due to the chain cross-linking reaction resulting from the formation of “inorganic silicon.” Furthermore, it was demonstrated that MCA was well embedded within the samples, and a cross-linking reaction occurred between the free radicals and oxygen under high temperatures, which significantly improved the thermal aging resistance. Apart from thermal aging resistance, effectively improving the tracking resistance and flame retardancy of SR is a key challenge in the development of advanced SR-based insulating materials.

**Figure 4:**
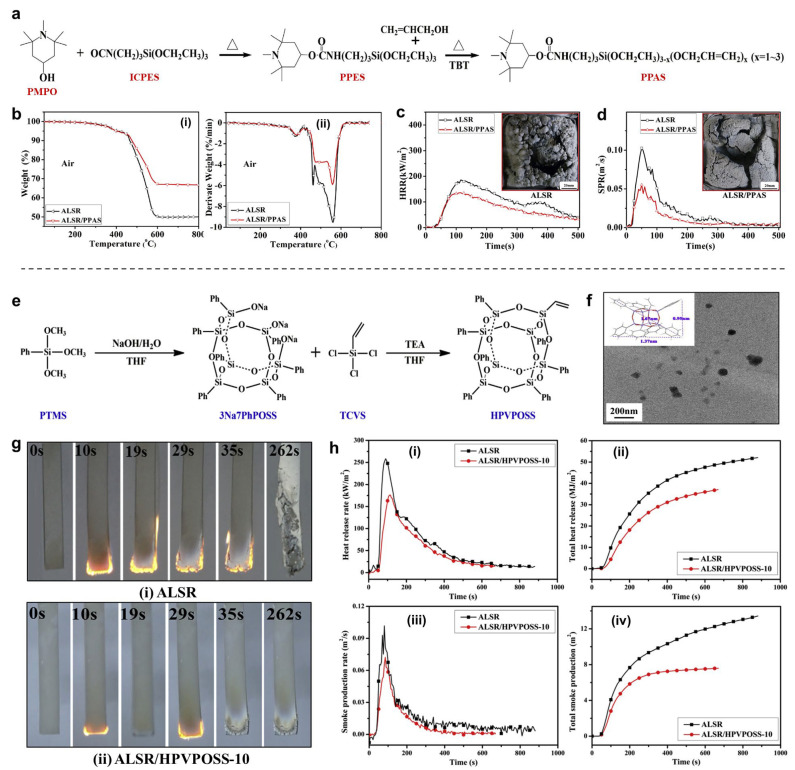
(**a**) Synthetic route of PPAS. (**b**) TGA (**i**) and DTG (**ii**) curves of the SR samples. (**c**) HRR and (**d**) SPR curves of the SR samples. Reproduced with permission from [24]. Copyright 2019, Elsevier. (**e**) Synthesis route of HPVPOSS. (**f**) TEM image of HPVPOSS. (**g**) UL-94 burning test of (**top**) ALSR and (**bottom**) ALSR/HPVPOSS-10. (**h**) Cone calorimeter test results of samples: (**i**) HRR, (**ii**) THR, (**iii**) SPR, and (**iv**) TSP curves. Reproduced with permission from [44]. Copyright 2019, Elsevier.

The Zeng group reported an addition-cure liquid SR with enhanced tracking resistance and good flame retardancy [24]. In this study, a functional silane (PPAS) containing hindered amine and urethane groups was synthesized through nucleophilic substitution and trans-etherification, as shown in Figure 4a,b. The results showed that PPAS can largely improve both the tracking resistance and flame retardancy of SR. With only 3.0 phr PPAS, the SR composite exhibited outstanding electrical erosion resistance and passed the 1A 4.5 kV level in the inclined plane test. In addition, it also possessed good self-extinguishing capability, with a LOI value of ~30.0%. Therefore, based on a series of chemical analyses, the possible mechanism was investigated and represented: PPAS had an effective quenching effect on peroxyl radicals, thereby restricting the oxidative degradation of Si-CH_3_ groups, eliminating Si-OH groups, and promoting the cross-linking re-action of SR during the thermal degradation process.

Silicon-containing flame retardants with low toxicity are environmentally friendly. Polymers with silicon-based flame retardants usually can generate SiO_2_ nanoparticles during their combustion process. The low surface energy allows such nano-silica to deposit onto the surface of the polymer, along with other decomposition products, to form a hybrid ceramic layer [44,67].

This protective layer can restrict the transfer of heat and combustible gas and oxygen, which can further protect the internal polymer from further thermal degradation and burning [68]. Polyhedral oligomeric silsesquioxanes (POSS), a type of silicon-based flame retardant, are nanosized cage-like hybrid molecules comprising silicon and oxygen with a rigid hollow silica core. This structure contributes to the flame retardancy, thermal stability, and anti-oxidation of polymers [69]. The size of POSS ranges from 1 to 3 nm, making them the smallest silica particles obtainable [70]. The functional groups present at the edges of the cage-like structure can be rationally designed to enhance the interfacial compatibility with polymers, allowing POSS to be incorporated as reactive components within the polymeric matrix [71]. To date, POSS and their derivatives have been applied to enhance the mechanical and thermal properties and reduce the fire hazards of various polymers, including SR [23,40,44,68,70,72].

Wu and co-authors synthesized hepta-phenyl vinyl polyhedral oligomeric silsesquioxane (called HPVPOSS) and incorporated it into SR to create an SR composite [44] (Figure 4e–h). HPVPOSS significantly improved both the flame retardancy and thermal stability of SR. With 10 phr HPVPOSS, the SR composite was able to achieve a UL-94V-0 rating and a LOI value of 31.0%. Additionally, there was a 31.8% decrease in the pHRR and a 28.7% decrease in THR compared to pure SR. More importantly, the SR/HPVPOSS nanocomposites exhibited good mechanical properties. The flame-retardant mechanism of HPVPOSS involves the quenching effect of free radicals and catalytic crosslinking charring of SR. Rybinski et al. designed and synthesized three different types of POSS-based flame-retardant compounds containing various groups—isobutyl and amino-propyl (AM-POSS), chloro-propyl (HA-POSS), and vinyl (OL-POSS)—for SR [68]. The effects of these POSS compounds, both individually and in synergy with melamine polyphosphate (MPP), on the thermal and flame-retardant properties of SR composites were studied. The results demonstrated effective improvements in the thermal properties and flame retardancy of SR. Furthermore, when MPP was used as a synergist with these POSS-based flame retardants, the OL-POSS/MPP system exhibited the highest flame-retardant efficiency.

**Figure 5:**
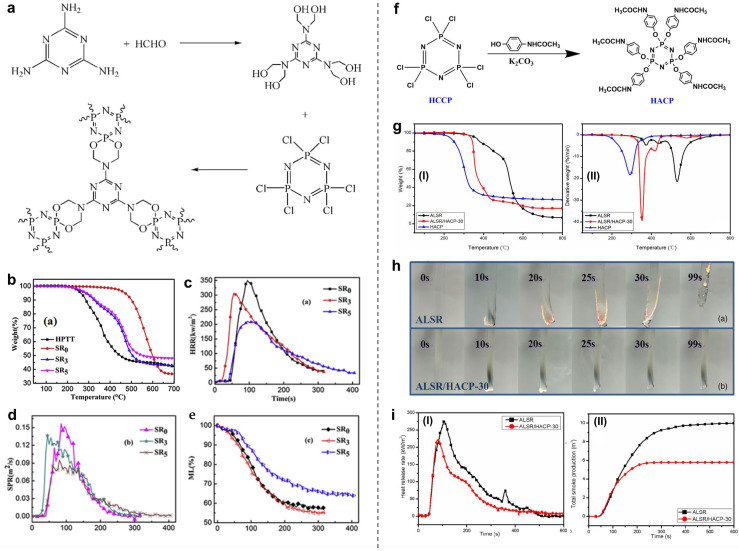
(**a**) The synthesis route of HPTT. (**b**) TG curves of HPTT, SR, and SR/HPTT systems. (**c**) HRR, (**d**) SPR, and (**e**) ML curves of SR and SR/HPTT composites. Reproduced with permission from [73]. Copyright 2015, Elsevier. (**f**) Synthesis route of HACP. (**g**) TGA and DTGA curves of samples under nitrogen atmosphere. (**h**) Vertical burning test of (**top**) ALSR and (**bottom**) ALSR/HACP-30. (**i**) HRR (**I**) and (**II**) TSP curves of ALSR and ALSR/HACP-30. Reproduced with permission from [77]. Copyright 2020, Wiley.

Currently, phosphorus–nitrogen flame retardants are the most commonly and widely used systems for achieving highly efficient flame retardancy in polymers. These flame retardants generally provide combined gas- and condensed-phase flame retardancy [64,73,74,75,76]. They offer advantages such as reduced smoke production, low toxicity, and anti-dripping properties [21]. Wang’s group designed and synthesized a P-N flame retardant (HPTT) using melamine (MA) and hexachlorocyclotriphosphazene (HCCP) as raw materials and applied it to fabricate flame-retardant SR [73], as illustrated in Figure 5a. The test results of the flame retardant demonstrated that, with the addition of 18 wt.% HPTT, the SR/HPTT composite could obtain a UL-94 V-0 rating and an LOI value of ~31.8%. Furthermore, its thermal stability also improved, with significant decreases in the HRR, pHRR, and SPR when compared to pure SR.

Yang and co-authors synthesized an efficient flame retardant, Hexa(p-acetamidophenoxy) cyclotriphosphazene (HACP), by using a simple one-step reaction method [77]. The HACP was then incorporated into SR to fabricate highly flame-retardant SR composites (Figure 5f–i). The introduction of HACP was found to effectively improve the flame resistance of SR. With the addition of 30 phr HACP, the SR composites achieved a UL-94 V-0 rating, and the THR and TSR decreased by approximately 27% and 42%, respectively. Furthermore, the test results confirmed the key role of HACP in forming protective layers and capturing free radicals.

#### 4.2.2. Inorganic Flame Retardants

The inorganic flame retardants achieve effective flame retardancy by reducing the proportion of combustible products and altering the thermal conductivity and other properties of polymer matrices. In terms of SR, the most widely used inorganic flame retardants include metal hydroxide, layered double hydroxide (LDH), metallic oxide, etc. [26,28,65,78].

Metal hydroxides, e.g., magnesium hydroxide (MH), are commonly used as flame-retardant fillers for various polymers due to their low cost and nontoxic smoke [25]. They can reduce the concentration of O_2_, absorb heat, and promote the formation of a protective char layer. Unfortunately, in practical application, to achieve adequate flame retardancy of polymers, high loading is needed due to their low flame-retardant efficiency. This would greatly damage the mechanical properties of SR. Therefore, it is vital to enhance the adhesion and dispersion of metallic hydroxides in SR to enhance their flame-retardant efficiency and overall performance.

Room-temperature vulcanized (RTV) SR has been widely applied as electrical insulation material. SR-filled ATH generally has good electrical insulation, but its flame retardancy is never very satisfying. Nazir prepared the ATH-filled SR, assisted by graphene nanoplatelets, and investigated its flame retardancy and electrical insulation performance [2]. The results indicated that the introduction of graphene nanoplatelets into the ATH-filled SR significantly enhanced the flame retardancy whilst maintaining the electrical insulation properties at an acceptable level. Fang et al. reported that magnesium hydroxide sulfate hydrate (MHSH) whiskers largely improve the flame retardancy of SR. Furthermore, the authors also found that the microencapsulated red phosphorus (MRP), when employed as an effective synergist, can further enhance the flame retardancy of the SR/MHSH composites [27]. Typically, upon adding 7 phr MRP, the SR composite sample has a higher LOI value of ~38.5% and can achieve a UL-94 V-0 rate. However, mechanical tests indicated that MHSH whiskers slightly decreased the tensile strength of the composites.

**Figure 6:**
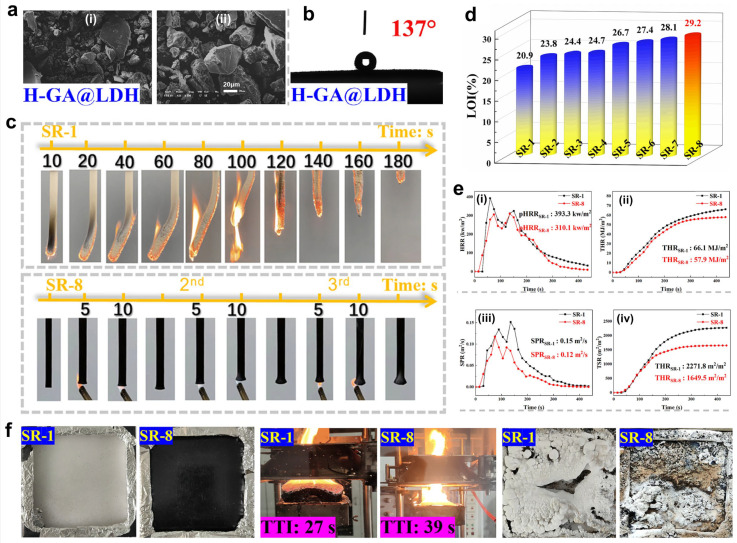
(**a**) SEM images and (**b**) WCA of H-GA@LDH. (**c**) UL-94 tests of SR-1 and SR-8 samples. (**d**) LOI vales of various samples. (**e**) Cone calorimeter results of samples: (**i**) HRR, (**ii**) THR, (**iii**) SPR, and (**iv**) TSR curves versus time. (**f**) Optical photos of combustion process of samples. Reproduced with permission from [78]. Copyright 2023, Elsevier.

**Figure 7:**
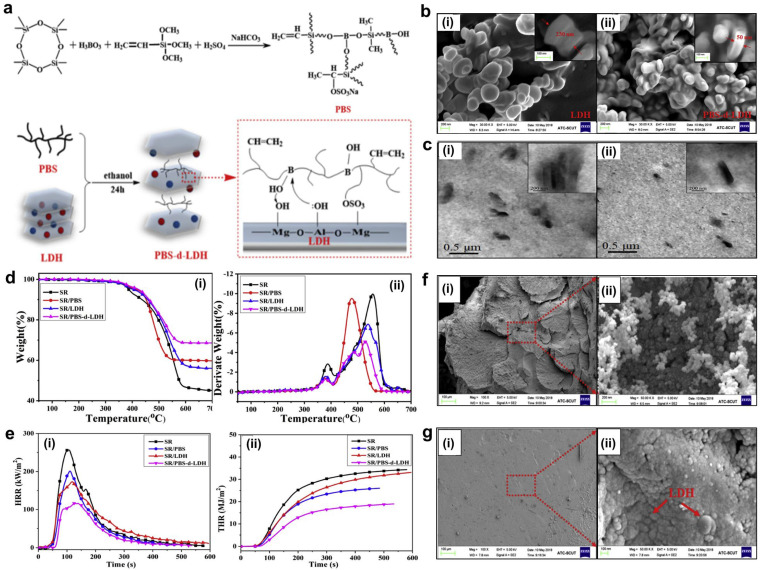
(**a**) Preparation scheme of PBS-d-LDH. (**b**) SEM images of LDH (**i**) and PBS-d-LDH (**ii**). (**c**) TEM images of SR/LDH (**i**) and SR/PBS-d-LDH (**ii**). (**d**) TGA curves of the SR samples under air atmosphere. (**e**) Cone calorimeter test data of samples: (**i**) HRR and (**ii**) THR curves versus time. SEM images of the char layer for SR (**f**) and SR/PBS-d-LDH (**g**) after cone calorimeter test. Reproduced with permission from [82]. Copyright 2019, Elsevier.

Layered double hydroxide (LDH) is one kind of eco-friendly flame-retardant filler with a layered crystalline structure. It can not only dilute the combustible gases through the release of CO_2_ and H_2_O, but also plays a role as a physical barrier to isolate flame and absorb smoke particles, thus effectively enhancing the flame retardancy and smoke suppression of the matrix [79,80,81]. Chai and co-authors utilized a natural product, Gum Arabic (GA), to modify LDH and prepared the H-GA@LDH; then, a novel and effective flame retardant for SR was obtained by combining H-GA@LDH with the synergist MWCNT [78] (Figure 6). As a result, with 1 wt.% MWCNT and 6 wt.% H-GA@LDH, SR composites were able to achieve a UL-94 V-0 rating, and the LOI value reached ~29.4%. In addition, pHRR and THR decreased by 22.14% and 12.43%, respectively. More importantly, such an SR system possesses good mechanical properties, flame retardancy, and water resistance, showing promising application prospects for fireproof cables in the field of high-voltage transmission. To achieve good dispersion of LDH in SR and further improve its flame-retardant efficiency, Qiu et al. designed and synthesized the polyborosiloxane-modified LDH (PBS-d-LDH) and added it into SR to fabricate SR composites [82] (Figure 7). Due to the presence of sulfonate and boron hydroxyl groups in PBS, PBS-d-LDH can be easily exfoliated and effectively dispersed in the SR matrix. The test results showed that PBS-d-LDH improved the mechanical properties, thermal stability, flame retardancy, and smoke suppression of SR. Typically, with only 5.0 phr PBS-d-LDH, the SR composite demonstrated good self-extinguishing capability, and the pHRR and pSPR decreased by 54.4% and 61.5%, respectively. Additionally, the char residue at 700 °C increased by 23.5%. The related results demonstrated that the synergy between the PBS crosslinking reaction and the LDH barrier effect enhanced the ceramization of the condensed phase, leading to enhanced flame retardancy and smoke suppression.

Metallic oxides are commonly used to enhance the thermal stability of polymers; however, they usually incorporate other synergist FRs to obtain good flame retardancy. Chen et al. explored the use of an intumescent flame retardant (composed of phosphorus acid, melamine, and pentaerythritol) and Fe_2_O_3_ as a smoke suppression agent in SR composites [28]. The results indicated that Fe_2_O_3_ can effectively enhance the smoke suppression effect and increase the thermal degradation temperature. Fe_2_O_3_ promotes the early cross-linking reaction of the polymer during decomposition, leading to increased char formation. The integrity of the nano-silica layer determines the physical barrier efficiency, as it can act as an ideal physical barrier by blocking heat transfer and external oxygen, thereby restricting further combustion of the inner polymer matrices. The combined catalysts not only increased the char yield of SR, but also improved the structure of its carbonaceous layer, significantly enhancing the flame retardancy and smoke suppression of SR composites. In another study, Huang et al. fabricated SR composites using Sb_2_O_3_ and melamine cyanurate (MCA) [65]. The SEM results showed that Sb_2_O_3_ and MCA were uniformly dispersed in the SR matrix. The test results indicated that the composite achieved a LOI of 31.5%, and the HRR and THR values of the composite with MCA were significantly reduced compared to the pure one. Additionally, the composite maintained good elongation and tensile strength.

#### 4.2.3. Intumescent Flame Retardants

In general, the intumescent flame-retardant system typically consists of three components, i.e., acid source, carbonizing agent, and blowing agent [64,83,84,85,86]. The acid source is an inorganic acid or a compound that generates acid during combustion. The carbonizing agent is a carbon-rich component that can be dehydrated by the catalytic action of the formed acid, accompanied by cross-linking and carbonization; the blowing agent can decompose and release nonflammable gas once it reaches a fixed temperature. Many researchers have conducted many studies on SR materials with intumescent flame retardants [87,88,89,90,91,92].

**Figure 8:**
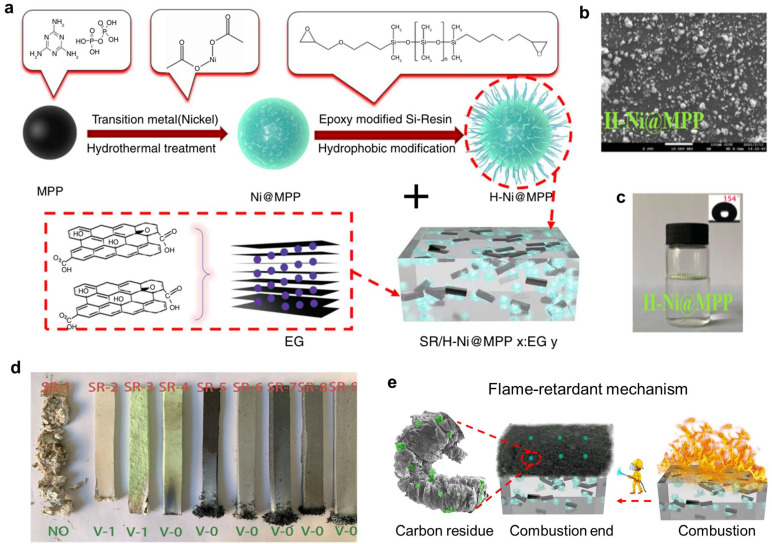
(**a**) Schematic diagram of the preparation of SR/H-Ni@MPPx/EGy. (**b**) SEM image and (**c**) digital image (inset is WCA test) of H-Ni@MPP. (**d**) Digital photos of char residues for samples after vertical burning tests and corresponding UL-94 grades. (**e**) Flame-retardant mechanism of SEM images of carbon residue of the SiF0 and SiF7. (**e**) Proposed flame-retardant mechanism. Reproduced with permission from [90]. Copyright 2023, Springer.

Hong and co-authors studied the effect of expanded graphite (EG) on the flame retardancy of SR [87]. The results showed that adding 25 phr of EG resulted in an LOI value of approximately 25%, and a UL-94 V-2 rating could be achieved. Additionally, the study also explored the impact of EG loading and size on the mechanical properties and flame retardancy of SR composites. Li et al. employed the intumescent flame retardant, i.e., melamine phosphate (MP), synergist nanofiller organic nano-montmorillonite (OMMT) to fabricate RTVSR/MP/OMMT composites [88]. The results showed that, with 35 phr MP and 10 phr OMMT, the SR nanocomposites showed the best comprehensive performance. Chai and co-authors successfully obtained a modified melamine polyphosphate (H-Ni@MPP) by introducing Ni^2+^ and epoxy-modified silicone resin [90] (Figure 8). Meanwhile, EG was used to further enhance the flame retardancy of SR. With the 40 phr H-Ni@MPP/EG, the SR composite was able to achieve a UL-94 V-0 rating; meanwhile, its LOI value reached up to ~50%. Moreover, it also displayed good water resistance due to hydrophobic modification. Based on the systematical analysis of the residue, it was demonstrated that the improved flame retardancy could be ascribed to the synergy effect of H-Ni@MPP and EG, the formation of a graphitized char layer, and the Ni-catalyzed effect.

#### 4.2.4. Nano-Flame Retardants

Nano-flame-retardant additives have demonstrated their unique advantages, making them a focal point of recent research in synergistic flame retardation. Numerous studies suggest that polymer nanocomposites hold great promise as the most effective flame-retardant materials for future applications [35,36,46,52,93,94,95,96]. Generally, a low loading of nanofillers can significantly enhance the flame retardancy of polymers, particularly by reducing the release of heat and smoke. Additionally, nano-flame retardants (fillers) are superior to most current flame retardants in terms of effectively improving the physical properties of polymer matrices. In terms of high-performance flame-retardant SR nanocomposites, the most typical nanomaterials include montmorillonite (MMT) [88,97,98,99], hexagonal boron nitride (h-BN) [31,100,101], graphene [102], carbon nanotubes [32,103], halloysite nanotubes (HNT) [33], etc. For instance, the low thermal conductivity of SR generally restricts their further application. To address these issues, researchers have introduced certain amounts of h-BN into polymers to improve both flame retardancy and thermal conductivity. Two-dimensional hexagonal boron nitride (2D-hBN) has a structure similar to graphene, making BN nanosheets possess excellent anti-oxidation. Furthermore, hBN sheet networks can create effective, high-thermal-conductivity pathways within the polymer matrices. Thus, they can effectively enhance the flame retardancy, thermal stability, as well as thermal conductivity of polymers [104,105,106].

**Figure 9:**
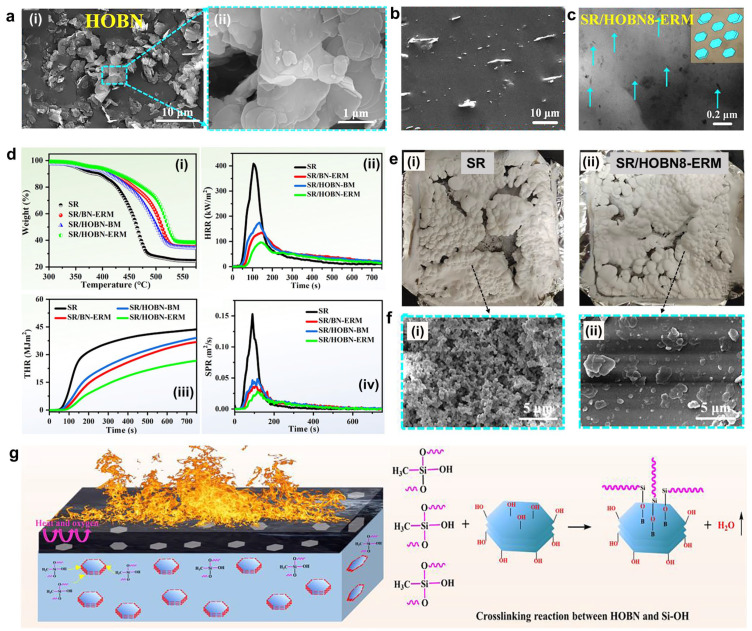
(**a**) SEM images of HOBN. (**b**) SEM image and (**c**) TEM image of SR/HOBN8-ERM nanocomposites. (**d**) TG and cone calorimeter test results of samples: (**i**) TG, (**ii**) HRR, (**iii**) THR, and (**iv**) SPR curves. (**e**) The digital photograph and (**f**) SEM images of the char layer after CCT for (**i**) SR and (**ii**) SR/HOBN8-ERM. (**g**) Proposed flame retardation mechanism of HOBN on SR. Reproduced with permission from [101]. Copyright 2022, Elsevier.

Wu et al. developed flame-retardant SR nanocomposites by incorporating hydroxylated BN (HOBN) using an eccentric-rotor internal mixer [101]. This mixer enhances hydrogen bonding between SR and HOBN through transient normal stress, thus promoting efficient exfoliation, homogeneous dispersion, and horizontal orientation of HOBN in the SR matrix. As shown in Figure 9, the SR/HOBN nanocomposite demonstrated outstanding flame retardancy, e.g., decreases of 76.4% in pHRR and 81.7% in SPR compared to pure SR. The study found that the barrier effect of HOBN and the cross-linking reaction between SR and HOBN during combustion created a synergistic effect by ceramizing the condensed phase. This process formed a compact and strong protective layer, greatly enhancing the smoke suppression and flame retardancy of SR.

**Figure 10:**
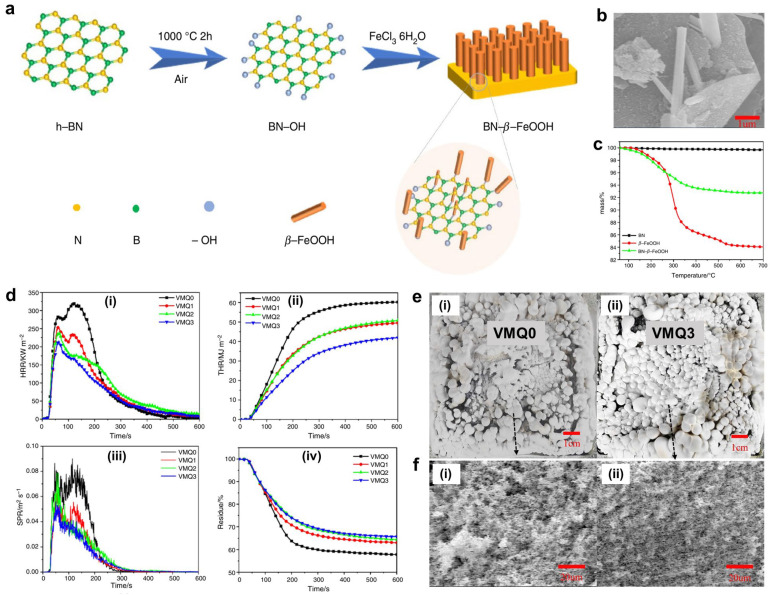
(**a**) Synthetic route of BN-β-FeOOH. (**b**) SEM image of BN-β-FeOOH. (**c**) TGA curves of as-prepared samples. (**d**) Cone calorimeter test results of VMQ composites samples: (**i**) HRR, (**ii**) THR, (**iii**) SPR, and (**iv**) char residue mass curves versus time. (**e**) Digital photos and (**f**) SEM images of the residues of (**i**) VMQ0 and VMQ3 after cone calorimetry tests. Reproduced with permission from [107]. Copyright 2023, Springer.

To achieve good flame retardancy and high thermal conductivity of SR, Xu and co-authors obtained a modified BN decorated by β-FeOOH (BN-β-FeOOH) [107]. BN-β-FeOOH was then incorporated into SR, and the effects of BN-β-FeOOH on the combustion behaviors and thermal conductivity of SR were studied (Figure 10). The test results indicated that the flame retardancy of SR was effectively enhanced, i.e., the LOI value of SR with 3 phr BN-β-FeOOH increased to 29.8%. Furthermore, the pHRR and THR were reduced by ~34.5 and ~30.2%, respectively. The improved flame retardancy of SR was mainly due to the physical barrier of BN and the catalytic carbonization of β-FeOOH. Additionally, adding BN-β-FeOOH significantly enhanced the thermal conductivity of SR. Specifically, adding 3 phr BN-β-FeOOH increased the thermal conductivity by 15.2% compared to neat SR. This improvement was primarily due to the in situ growth of β-FeOOH on the BN surface, creating a well-connected thermal network, thus effectively improving the thermal conductivity of SR.

**Figure 11:**
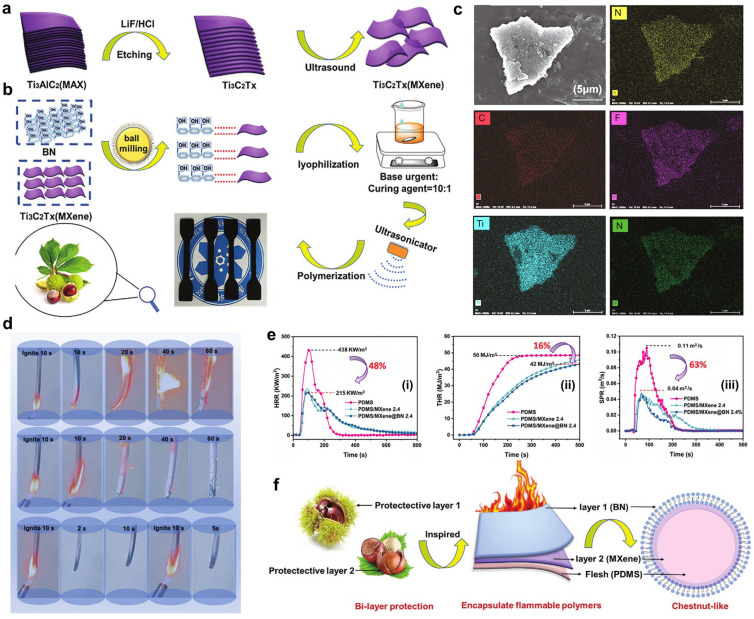
(**a**) Exfoliating process of MXene and (**b**) the fabrication of PDMS/MXene@BN composites. (**c**) SEM image of MXene@BN and corresponding EDS mapping images. (**d**) Burning testing of pure PDMS, PDMS/MXene, and PDMS/MXene@BN composites. (**e**) Cone calorimeter test results of composite samples: (**i**) HRR, (**ii**) THR, and (**iii**) SPR curves versus time. (**f**) Proposed flame-retardant mechanism. Reproduced with permission from [31]. Copyright 2023, Wiley.

Silicon-based elastomers with enhanced thermal conductivity, flame retardancy, and electromagnetic shielding are in high demand in the electronics field. Bian et al. developed a simple and eco-friendly ball milling shear method to produce MXene@Boron nitride (MXene@BN), which was then introduced into polydimethylsiloxane (PDMS) to create a multifunctional PDMS/MXene@BN composite with excellent electromagnetic interference (EMI) shielding, thermal conductivity, and flame retardancy [31] (Figure 11). Benefiting from the exceptional chestnut-like structure of MXene@BN, the thermal conductivity of composite could reach up to ~0.6 W m^−1^ K^−1^, which was twice as high as that of the neat PDMS. In addition, the TSP value of the composite was ~64% lower than the neat PDMS. Furthermore, the EMI SE of the PDMS composite could reach 26.3 dB at 8.5 GHz. The excellent comprehensive performance indicates its promising application prospects as a high-performance flexible electronic packaging material.

#### 4.2.5. Other Flame Retardants

Apart from the aforementioned flame-retardant system, other types of flame retardants and hybrid flame-retardant systems have also been investigated. Platinum (Pt) or platinum compounds, as high-temperature catalysts, are highly effective in flame-retardant SR. They can enhance the cross-linking density and increase the char residue of SR, thus improving its flame-retardant properties. Chen et al. combined Pt catalysts with nitrogen-containing silane (NS) to obtain SR composites with improved thermal stability [108]; the possible mechanism can be attributed to the improved catalytic efficiency due to the nitrogen atoms coordinated with Pt. Furthermore, NS could effectively preserve the high catalytic activity of Pt. Thus, Pt/NS catalyzed the crosslinking reaction and restricted the degradation of SR chains. Furthermore, the related results indicated that the introduction of Pt/NS can protect SR chains from oxidation. In addition, Liu and co-authors revealed that the Pt/N catalytic system can largely promote the organic-to-inorganic conversion of SR at the high-temperature stage [109]. Typically, with 0.33 phr Pt catalyst and 2 phr nitrogenous silane, the char residual rate of SR composite at 1000 °C was significantly improved from ~3.0% to ~45%. Jiang et al. prepared a ceramifiable SR composite by adding a complexing agent of Karstedt’s Pt catalyst and benzotriazole (Pt/BTA) to the SR containing aluminum hydroxide (ATH), ZrSiO_4_, and a glass dust system [110]. When the Pt/BTA content was ~1138 ppm, the SR composite was able to achieve a UL-94V-0 rating.

Su et al. employed the bio-based polyhydroxy compound cyclodextrin (CD) to obtain a hybrid intumescent flame-retardant system with ammonium polyphosphate (APP) to reduce the fire hazard of SR [61]. The results indicated that, with 40 phr total fillers, the LOI of SR composites could reach ~31%, and a UL-94 V-0 rating could also be obtained. In addition, benefiting from the surface modification of CD and APP, the water resistance and compatibility of SR composites were effectively enhanced. In practical applications of SR, especially for flame or high-temperature environments, it is required not only that SR materials possess good flame retardancy, but also that they have excellent structural stability. Therefore, fire-resistant ceramifiable SR composites with excellent comprehensive performance are highly attractive. Generally, the char residues of pure SR are fragile due to the formation of a porous nano-silica layer after combustion. Incorporating the ceramifiable fillers into SR is an effective way to achieve SR composites with good to excellent structural stability.

**Figure 12:**
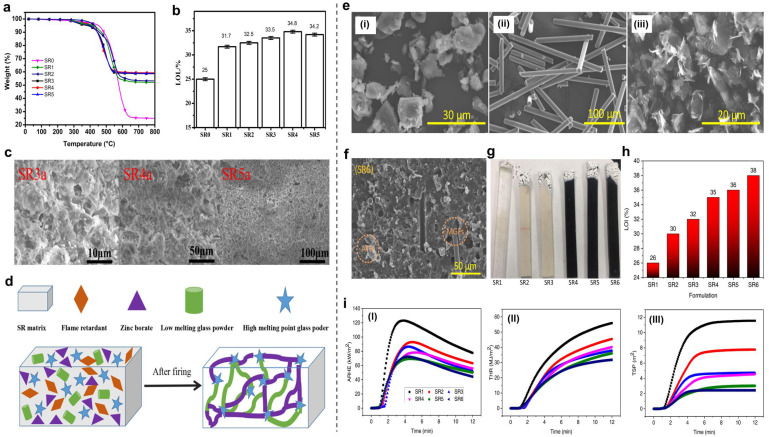
(**a**) TG results of samples under a nitrogen atmosphere. (**b**) The LOI values of different SR composites. (**c**) Cross-sectional SEM images of SR3 at 600 °C, showing the formation of ceramic structure. (**d**) Proposed ceramifying mechanism of SR composite. Reproduced with permission from [111]. Copyright 2020, Wiley. (**e**) Additives used for the synthesis of composites (**i**) ATH, (**ii**) MGFs, and (**iii**) GNPs. (**f**) Cross-sectional SEM image of SR6 composites. (**g**) Digital photos of samples after LOI test and (**h**) corresponding LOI values. (**i**) ARHE, THR, and TSP curves of SR composites. Reproduced with permission from [112]. Copyright 2022, Elsevier.

Li et al. designed and prepared a ceramifiable flame-retardant SR composite by introducing magnesium hydroxide, aluminum hydroxide, glass frits, and zinc borate into SR [111], as shown in Figure 12a–d. The ceramifying SR composite was able to achieve a LOI value of ~35%, and the flexural strength of ceramic residues reached up to 9.7 MPa. The residue of the SR composite was ~58.6% at 700 °C, which was largely higher than that of pure SR (25.8%). More importantly, the related chemical analysis and structural observation revealed the formation process of the dense ceramic structure of SR at high temperatures. Zinc borate plays a double role, i.e., as a flame retardant and a low-melting-point binder, in ceramic systems. Low-melting glass powder and magnesium hydroxide also play crucial roles in the ceramic-forming process. At 800 °C, zinc borate and the glass powder form a eutectic with amorphous silica, resulting in a eutectic reaction that creates a new crystal phase. This process produces coherent and compact microstructures at high temperatures, significantly improving the flexural strength of ceramic residues. Nazir and co-authors developed an SR composite for electrical insulation during wildfires. They introduced milled glass fibers (MGFs) and graphene nanoplatelets (GNPs) into a PDMS filled with aluminum trihydroxide (ATH) [112] (see Figure 12e–i). The results showed that adding MGFs and GNPs to the ATH-filled SR improved the flame retardancy, mechanical properties, and surface hydrophobicity without sacrificing the electrical breakdown voltage.

## 5. Conclusions and Outlooks

This review focuses on two predominant strategies for enhancing the flame retardancy of SR: intrinsic flame retardation and additive-type flame retardation. Intrinsic flame-retardant SR uniquely provides enduring flame retardancy while preserving mechanical properties. Nonetheless, the range of effective flame-retardant units is very limited, and the preparation process is complex; thus, it is difficult to industrialize. Additive-type flame retardation represents the conventional approach for fabricating flame-retardant SR materials, offering a wide array of flame retardants and fillers for integration into SR. This approach is supported by various synthetic and modification techniques that allow for the development of highly effective flame-retardant systems. Halogen-containing flame retardants have been gradually restricted and banned due to their environmental hazards, leading to a prominent trend of developing halogen-free flame-retardant polymeric materials. P-based and N-based additives enhance flame retardancy through phase and gas actions, yet individually, P, N, or Si-based flame retardants do not achieve ideal effectiveness. In contrast, P-N synergistic flame retardants exhibit high flame-retarding efficiency but suffer from complex, time-intensive synthesis and low yields. These additives also demonstrate poor compatibility with the SR matrix, adversely affecting the material’s mechanical properties. Therefore, the design and synthesis of P-N flame-retardant compounds must take into account the issue of compatibility. Inorganic flame retardants, such as metal hydroxides, layered double hydroxides, and metal oxides, offer environmental and cost benefits, but lack high flame-retardant efficiency. Achieving adequate flame retardancy in SR typically necessitates a substantial loading of these inorganic fillers, which significantly impairs the mechanical properties of the SR matrix due to poor interfacial compatibility. In addition, excess additives will increase the system viscosity and bring difficulties to the processing. Until now, the hybrid systems composed of P/N synergistic flame retardants and inorganic flame retardants have been regarded as the most promising flame-retardant systems for achieving highly flame-retardant SR composites. Platinum or platinum compounds have good catalytic efficiency, but the cost is high. Furthermore, their sole utilization cannot enable the SR to achieve a high LOI; thus, they should be combined with other additive flame retardants. Nano-flame-retardants provide low loading, high efficiency, and minimal impact on SR mechanical properties. However, preventing the formation of agglomerates and ensuring the uniform dispersion of nanomaterials within the matrix present significant challenges. Advancements in surface modifications to improve the compatibility between nanofillers and the matrix are critical. For advanced flame-retardant SR materials that require special properties, like high thermal conduction, electromagnetic shielding, and anti-ablation, functional nanofillers such as BN, graphene, MXene, and ceramic nanofillers are ideal candidates for achieving this. Despite significant progress, no perfect flame-retardant SR system yet exists, as each presents unique advantages and disadvantages. Future research should aim to enhance the positive aspects and mitigate the negative impacts of each system to create SRs with excellent overall performance. With the deepening of research, a single flame-retardant system is increasingly unable to fabricate high-performance flame-retardant SR, and synergistic flame-retardant systems will become more and more popular.

## Figures and Tables

**Table 1:** Criteria for UL94 flame-retardant ratings.

Criteria	No Rating	V-2	V-1	V-0
Maximum flame burning time for a single sample (t_1_/t_2_)	>30 s	≤30 s	≤30 s	≤10 s
Total flame combustion time of five samples (t_1_ + t_2_)	>250 s	≤250 s	≤250 s	≤50 s
Flame and no-flame burning time after second ignition (t_2_ + t_3_)	>60 s	≤60 s	≤60 s	≤30 s
Whether flaming or flame burning spreads to the fixture	No	No	No	No
Whether the dropping ignites the cotton wool	No	No	No	No

## Data Availability

Not applicable.
